# Sex-related differences in patients presenting with heart failure–related cardiogenic shock

**DOI:** 10.1007/s00392-024-02392-8

**Published:** 2024-02-14

**Authors:** Jonas Sundermeyer, Caroline Kellner, Benedikt N. Beer, Lisa Besch, Angela Dettling, Letizia Fausta Bertoldi, Stefan Blankenberg, Jeroen Dauw, Zouhir Dindane, Dennis Eckner, Ingo Eitel, Tobias Graf, Patrick Horn, Joanna Jozwiak-Nozdrzykowska, Paulus Kirchhof, Stefan Kluge, Axel Linke, Ulf Landmesser, Peter Luedike, Enzo Lüsebrink, Nicolas Majunke, Norman Mangner, Octavian Maniuc, Sven Möbius-Winkler, Peter Nordbeck, Martin Orban, Federico Pappalardo, Matthias Pauschinger, Michal Pazdernik, Alastair Proudfoot, Matthew Kelham, Tienush Rassaf, Clemens Scherer, Paul Christian Schulze, Robert H. G. Schwinger, Carsten Skurk, Marek Sramko, Guido Tavazzi, Holger Thiele, Luca Villanova, Nuccia Morici, Ephraim B. Winzer, Dirk Westermann, Benedikt Schrage

**Affiliations:** 1 Department of Cardiology, University Heart and Vascular Center Hamburg, Martinistr. 52, 20251 Hamburg, Germany; 2 German Center for Cardiovascular Research (DZHK), Partner Site Hamburg/Kiel/Lübeck, Hamburg, Germany; 3 Cardio Center, Humanitas Clinical and Research Center - IRCCS, Rozzano, Milan Italy; 4 Center for Population Health Innovation (POINT), University Heart and Vascular Center Hamburg, University Medical Center Hamburg-Eppendorf, Hamburg, Germany; 5 Department of Cardiology, AZ Sint-Lucas, Ghent, Belgium; 6 Department for Internal Medicine and Cardiology, Heart Centre Dresden, University Hospital, Dresden, Germany; 7 Department of Cardiology, Paracelsus Medical University Nürnberg, Nuremberg, Germany; 8 University Heart Center Lübeck, University Hospital Schleswig-Holstein, Lübeck, Germany; 9 Division of Cardiology, Pulmonology and Vascular Medicine, Medical Faculty, University Duesseldorf, Düsseldorf, Germany; 10 Department of Internal Medicine and Cardiology, Heart Center Leipzig at University of Leipzig and Leipzig Heart Science, Leipzig, Germany; 11 Department of Intensive Care Medicine, University Medical Center Hamburg-Eppendorf, Hamburg, Germany; 12 Department of Cardiology, Angiology and Intensive Care Medicine, DHZC Berlin, Campus Benjamin Franklin, Berlin, Germany; 13 Department of Cardiology and Vascular Medicine, West German Heart and Vascular Center, University Hospital Essen, Essen, Germany; 14 Department of Medicine I, University Hospital, LMU Munich, Munich, Germany; 15 Department of Internal Medicine I, University Hospital Würzburg, Würzburg, Germany; 16 Department of Internal Medicine I, University Hospital Jena, Jena, Germany; 17 Dept Cardiothoracic and Vascular Anesthesia and Intensive Care, AO SS Antonio E Biagio E Cesare Arrigo, Alessandria, Italy; 18 Department of Cardiology, IKEM, Prague, Czech Republic; 19 Department of Perioperative Medicine, St. Bartholomew’s Hospital, London, UK; 20 Medizinische Klinik II, Kliniken Nordoberpfalz AG, Weiden, Germany; 21 Department of Clinical-Surgical, Diagnostic and Paediatric Sciences, Anesthesia and Intensive Care, University of Pavia Italy, Fondazione Policlinico San Matteo Hospital IRCCS, Pavia, Italy; 22 Unità Di Cure Intensive Cardiologiche and De Gasperis Cardio-Center, ASST Grande Ospedale Metropolitano Niguarda, Milan, Italy; 23 IRCCS Fondazione Don Gnocchi, ONLUS, Santa Maria Nascente, Milan, Italy; 24 Department of Cardiology and Angiology, University Heart Center Freiburg-Bad Krozingen, Freiburg, Germany

**Keywords:** Cardiogenic shock, Heart failure, Sex disparities, Gender differences, Women in heart failure

## Abstract

**Background:**

Heart failure–related cardiogenic shock (HF-CS) accounts for a significant proportion of all CS cases. Nevertheless, there is a lack of evidence on sex-related differences in HF-CS, especially regarding use of treatment and mortality risk in women vs. men. This study aimed to investigate potential differences in clinical presentation, use of treatments, and mortality between women and men with HF-CS.

**Methods:**

In this international observational study, patients with HF-CS (without acute myocardial infarction) from 16 tertiary-care centers in five countries were enrolled between 2010 and 2021. Logistic and Cox regression models were used to assess differences in clinical presentation, use of treatments, and 30-day mortality in women vs. men with HF-CS.

**Results:**

*N* = 1030 patients with HF-CS were analyzed, of whom 290 (28.2%) were women. Compared to men, women were more likely to be older, less likely to have a known history of heart failure or cardiovascular risk factors, and lower rates of highly depressed left ventricular ejection fraction and renal dysfunction. Nevertheless, CS severity as well as use of treatments were comparable, and female sex was not independently associated with 30-day mortality (53.0% vs. 50.8%; adjusted HR 0.94, 95% CI 0.75–1.19).

**Conclusions:**

In this large HF-CS registry, sex disparities in risk factors and clinical presentation were observed. Despite these differences, the use of treatments was comparable, and both sexes exhibited similarly high mortality rates. Further research is necessary to evaluate if sex-tailored treatment, accounting for the differences in cardiovascular risk factors and clinical presentation, might improve outcomes in HF-CS.

**Graphical abstract:**

Sex-related differences in clinical characteristics, shock severity, and mortality in patients with heart failure–related cardiogenic shock. Summary for the main study findings. AMI, acute myocardial infarction; CI, confidence interval; HF-CS, heart failure–related cardiogenic shock; LVEF, left ventricular ejection fraction; MCS, mechanical circulatory support; SCAI, Society for Cardiovascular Angiography & Interventions.

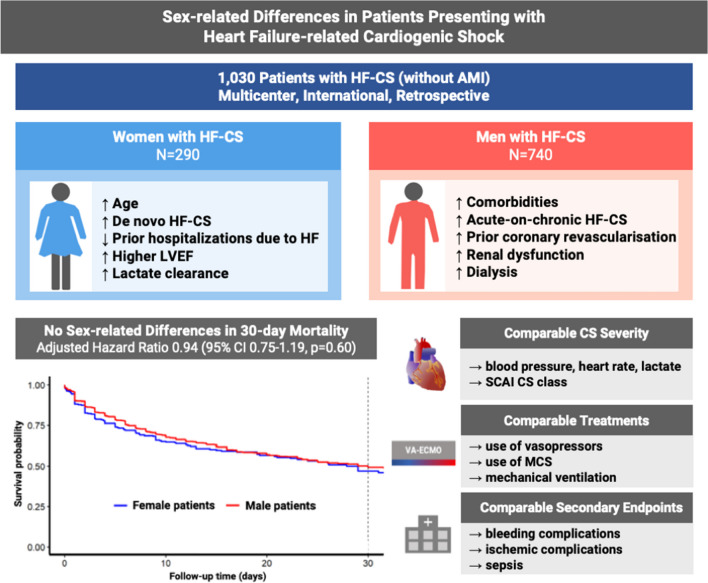

## Introduction

Heart failure–related cardiogenic shock (HF-CS) is a severe condition characterized by acute deterioration in cardiac output that results in a life-threatening hypoperfusion of end-organs. This condition can be caused by a variety of cardiovascular diseases and has been associated with short-term mortality rates of up to 50% [1–6]. While research efforts have predominantly focused on CS due to acute myocardial infarction (AMI-CS), recent studies suggest that more than half of all cases of CS may be related to HF [1–8]. Despite significant advances in the management of AMI-CS due to early coronary revascularization, therapeutic strategies for HF-CS remain insufficiently understood, particularly with respect to targeted use of inotropics, vasopressors, mechanical circulatory support (MCS), and cause-specific therapeutic modalities [7, 9–13].

The management of HF-CS is complicated by the heterogeneity of underlying pathologies. The lack of clinical trials and recommendations in the current guidelines further exacerbates the clinical uncertainty in the treatment of HF-CS, particularly with regard to the extent of differences in clinical presentation and response to treatment modalities within this heterogeneous population of shock patients [11, 14, 15]. Moreover, there is a scarcity of data regarding sex-related differences in patients presenting with CS, particularly with HF-CS. Current findings originate from small randomized trials or retrospective analyses that have limited representation of women, and predominantly focus on sex disparities in patients with AMI-CS [16–19]. Women with AMI-CS tend to be older, have a higher burden of comorbidities, receive fewer MCS, and experience higher in-hospital mortality, compared to men [16–20].

Optimizing care in HF-CS requires understanding sex differences in pathophysiology and clinical presentation. Additionally, recognizing the influence of sex on treatment response and targeted interventions has the potential to improve outcomes and provide personalized care to both male and female patients with HF-CS. Enhancing comprehension of sex disparities could not only aid in the advancement of effective treatment approaches but also promote the inclusion and representation of women in clinical trials within the field of patients with CS.

Thus, the objective of this study is to investigate sex-related differences in clinical presentation, treatment modalities, and outcome in a large cohort of patients with HF-CS.

## Methods

### Data source and setting

The current study was carried out in conformity with the tenets of the Declaration of Helsinki and received endorsement from local ethics committees. The principal ethics committee granted an exemption for obtaining informed consent due to the retrospective nature of the study, which solely relied on fully anonymized data.

Comprehensive documentation regarding the data entry process, definition of CS, and the criteria for inclusion and exclusion in the non-ischemic cardiogenic shock registry (NCT03313687) has been described [21–23]. In brief, in this concise international, multicenter, observational investigation, we retrospectively included patients diagnosed with HF-CS (without AMI) from 16 tertiary care centers across five countries during the period spanning 2010 to 2021. The patients received treatment either with or without MCS devices, and each tertiary care center possessed extensive expertise in MCS application, along with the provision of percutaneous left ventricular assist devices (Impella) and veno-arterial extracorporeal membrane oxygenation (VA-ECMO). Patients were considered eligible for participation in this research if they fulfilled the criteria for CS as defined by the Society for Cardiovascular Angiography & Interventions (SCAI) [2]. The necessary data were retrospectively collected by the local investigators subsequent to a comprehensive review of the available case records.

### Definition of HF-CS

The local investigators identified patients either as presenting with a first manifestation of HF-CS without a history of HF, also known as de novo HF-CS, or as presenting with an acute-on-chronic HF-CS, such as patients with a known history of HF. Patients who met any of the following criteria were excluded from participation in this registry: presentation with acute myocardial infarction (AMI) or requirement for urgent coronary revascularization, regardless of feasibility; CS primarily caused by right heart failure (such as acute pulmonary embolism); cardiopulmonary resuscitation (CPR) supported by ECMO; post-cardiotomy CS; or other illnesses resulting in a life expectancy of less than 6 months.

### Index event definition

In cases involving patients who underwent treatment with MCS, the index event was defined as the time of initial device implantation. Conversely, for patients who did not receive MCS therapy, the baseline was established as their hospital admission for out-patients or their admission to the intensive care unit for in-hospital patients. To evaluate the severity of CS, the most extreme value of certain laboratory parameters, such as lactate and pH levels, within a 12-h timeframe encompassing 6 h before and after the index event was recorded.

### Outcome definition

The primary endpoint of the study was the cumulative mortality rate at 30 days. As for the secondary endpoints, evaluation included bleeding complications, ischemic complications, the need for renal replacement therapy, and the occurrence of sepsis.

### Statistical analyses

Continuous variables are presented as median (25th percentile, 75th percentile) and groups were compared using the Mann–Whitney test. For binary variables, absolute and relative frequencies were reported, and comparisons were performed using Fisher’s exact test.

To investigate differences in clinical characteristics during the index event, comorbidities, shock severity (SCAI risk stratification), use of selected treatments (vasopressors, MCS use), and complications between female and male patients with HF-CS, multivariable mixed effects logistic regression models with center as a random intercept were fitted, adjusted for age, SCAI class, lactate, pH, prior CPR, and mechanical ventilation.

To evaluate CS dynamics following the index event, trajectories of lactate levels (as an indicator of shock severity) and creatinine levels (as a marker of end-organ damage) in female and male patients diagnosed with HF-CS over a period of 7 days from baseline were displayed and comparatively analyzed using the Kruskal–Wallis test.

The reverse Kaplan–Meier estimator was used to calculate the crude 30-day all-cause mortality rate in women vs. men with HF-CS. Survival curves were estimated using the Kaplan–Meier method and the number of individuals at risk was reported. To evaluate the association between women and men and the primary outcome of 30-day all-cause mortality, adjusted Cox regression models were fitted, adjusted for age, SCAI class, lactate, pH, prior CPR, and mechanical ventilation. Odds ratios (OR) and 95% confidence intervals (CI) are presented, a *p* value below 0.05 was considered statistically significant. Analyses were performed using R statistical software version 4.1.2.

## Results

### Study cohort

Overall, 1030 patients with HF-CS were enrolled in this registry, and all patients were eligible for the study, of whom 290 (28.2%) were women and 740 (71.8%) were men (***Graphical abstract***). Baseline characteristics for the overall cohort stratified by women vs. men with HF-CS are shown in Table 1.

**Table 1:** Characteristics for the overall cohort and stratified by sex

	All (*N* = 1030)	Missing data (%)	Female patients (*N* = 290)	Male patients (*N* = 740)	*p* value
Demographics					
Age, years	64.0 (52.0, 75.0)	0	69.0 (54.0, 79.0)	63.0 (52.0, 72.0)	< 0.001
Previous heart failure status					
Ischemic cardiomyopathy	247 (34.0)	29.4	40 (23.5)	207 (37.2)	0.001
Previous heart failure hospitalizations, *n*	2.0 (1.0, 3.0)	67.2	1.0 (1.0, 2.0)	2.0 (1.0, 3.0)	0.009
Previous heart failure treatment					
Betablocker	543 (54.2)	2.8	138 (49.5)	405 (56.1)	0.066
Renin-angiotensin-system inhibitors	477 (47.6)	2.7	116 (41.4)	361 (50.0)	0.017
Mineralocorticoid receptor antagonists	327 (32.6)	2.6	68 (24.2)	259 (35.9)	< 0.001
Implantable cardioverter defibrillator	299 (29.1)	< 1	38 (13.1)	261 (35.3)	< 0.001
Cardiac resynchronization therapy	124 (12.1)	< 1	19 (6.6)	105 (14.2)	< 0.001
Comorbidities					
Atrial fibrillation	444 (44.2)	2.4	97 (35.0)	347 (47.7)	< 0.001
Diabetes mellitus	274 (27.0)	1.5	69 (24.3)	205 (28.0)	0.24
Arterial hypertension	582 (57.6)	1.9	151 (53.2)	431 (59.4)	0.077
Body mass index, kg/m^2^	26.2 (23.4, 30.0)	4.2	25.7 (22.0, 30.0)	26.6 (23.9, 30.1)	0.003
Prior coronary revascularization	250 (25.4)	4.6	43 (15.1)	207 (29.7)	< 0.001
Any intervention for peripheral artery disease	59 (5.8)	35	15 (5.3)	44 (6.0)	0.77
Clinical presentation					
Systolic blood pressure, mmHg (worst value within 6 h)	82.0 (70.0, 92.0)	1.9	80.5 (70.0, 95.0)	82.0 (70.8, 91.0)	0.99
Diastolic blood pressure, mmHg (worst value within 6 h)	50.0 (40.0, 57.0)	2.4	50.0 (40.0, 55.0)	50.0 (40.0, 58.0)	0.24
Vasopressor use	893 (86.8)	< 1	256 (88.3)	637 (86.2)	0.41
Heart rate, bpm (worst value within 6 h)	96.0 (76.0, 120.0)	1.7	96.0 (76.0, 120.0)	96.0 (78.0, 120.0)	0.50
Lactate, mmol/l (worst value within 6 h)	5.0 (2.7, 8.6)	8.9	5.2 (2.5, 8.8)	5.0 (2.7, 8.5)	0.91
pH (worst value within 6 h)	7.3 (7.2, 7.4)	4.4	7.3 (7.2, 7.4)	7.3 (7.2, 7.4)	0.94
LVEF (%)	20 (15.0, 30.0)	21.7	25.0 (20.0, 33.8)	20.0 (15.0, 30.0)	< 0.001
CPR	395 (38.6)	< 1	115 (39.8)	280 (38.1)	0.62
CPR > 10 min	256 (55.8)	55.4	80 (59.7)	176 (54.2)	0.30
Mechanical ventilation	659 (65.2)	1.9	184 (64.3)	475 (65.6)	0.71
Horowitz index (worst value within 6 h)	189.2 (102.7, 289.0)	31.0	208.5 (128.8, 295.5)	176.7 (97.3, 281.0)	0.017
Creatinine, mg/dl (worst value within 6 h)	1.7 (1.2, 2.5)	1.9	1.4 (1.0, 2.1)	1.8 (1.3, 2.7)	< 0.001
SCAI cardiogenic shock class		3.0			
B	151 (15.1)		40 (14.4)	111 (15.4)	0.77
C	337 (33.7)		100 (36.0)	237 (32.9)	0.37
D	241 (24.1)		62 (22.3)	179 (24.8)	0.46
E	270 (27.0)		76 (27.3)	194 (26.9)	0.94
Most likely trigger					
Bradyarrhythmia	20 (3.0)		7 (3.8)	13 (2.7)	0.45
Tachyarrhythmia	216 (32.8)		49 (26.9)	167 (35.0)	0.051
Infection	111 (16.8)		29 (15.9)	82 (17.2)	0.73
Metabolic	16 (2.4)		4 (2.2)	12 (2.5)	1.00
Non-adherence to medical treatment	16 (2.4)		3 (1.6)	13 (2.7)	0.58
Postoperative	10 (1.5)		3 (1.6)	7 (1.5)	1.00
Stress	29 (4.4)		23 (12.6)	6 (1.3)	< 0.001
Toxic	19 (2.9)		6 (3.3)	13 (2.7)	0.79
No trigger	222 (33.7)		58 (31.9)	164 (34.4)	0.58
Use of mechanical circulatory support					
VA-ECMO only	169 (16.4)	0	36 (12.4)	133 (18.0)	0.031
Impella only	146 (14.2)	0	40 (13.8)	106 (14.3)	0.92
Impella + VA-ECMO	91 (8.8)	0	22 (7.6)	69 (9.3)	0.46
Use of antegrade perfusion cannula for VA-ECMO	199 (43.7)	0	46 (39.3)	153 (45.3)	0.28

Continuous variables are shown as a median (25th, 75th percentile), binary variables as absolute and relative frequencies, the *p* value given is calculated for continuous variables by Mann–Whitney test or binary variables by Fisher’s exact test. *CPR*, cardiopulmonary resuscitation; *LVEF*, left ventricular ejection fraction; *SCAI*, Society for Cardiovascular Angiography & Interventions; *VA-ECMO*, veno-arterial extracorporeal membrane oxygenation

The median age of the entire patient population was 64 (interquartile range (IQR) 52–75) years. Among the patients with HF-CS, 582 (57.6%) had a documented diagnosis of arterial hypertension, 274 (27.0%) had diabetes mellitus, and 444 (44.2%) had a history of atrial fibrillation. Furthermore, 247 (34.0%) patients had a previous history of ischemic cardiomyopathy, and 250 (25.4%) had undergone prior coronary revascularization (but no need for urgent coronary revascularization and no AMI during the shock index event).

At the index event, the baseline lactate level was 5.0 (IQR 2.7–8.6) mmol/l, and the baseline pH value was 7.3 (IQR 7.2–7.4). Patients presented with a systolic blood pressure of 82 (IQR 70.0–92.0) mmHg and a diastolic blood pressure of 50.0 (40.0–57.0) mmHg. The median baseline left ventricular ejection fraction (LVEF) of the study cohort was 20 (IQR 15–30) %. Overall, 395 (38.6%) patients underwent CPR, and 659 (65.2%) patients required mechanical ventilation, with a Horowitz index (PaO2/FiO2) of 189.2 (IQR 102.7–289.0).

### Sex-related differences in clinical presentation in HF-CS

Among patients with HF-CS, women tended to be older than men, even after adjusting for relevant confounders (OR 0.99, 95% CI 0.98–1.00, *p* = 0.037). Cardiovascular risk factors such as arterial hypertension (OR 1.68, 95% CI 1.21–2.35, *p* = 0.002) and diabetes (OR 1.42, 95% CI 1.00–2.02, *p* = 0.053), as well as cardiac comorbidities such as atrial fibrillation (OR 1.98, 95% CI 1.44–2.72, *p* < 0.001), were less prevalent in women. Moreover, women were less likely to have a known history of heart failure (OR 2.69, 95% CI 1.96–3.70, *p* < 0.001) and prior coronary revascularization (OR 2.64, 95% CI 1.77–3.94, *p* < 0.001). Furthermore, prior hospitalizations due to HF were less likely in women vs. men (OR 1.26% CI 1.02–1.57, *p* = 0.035).

**Fig. 1:**
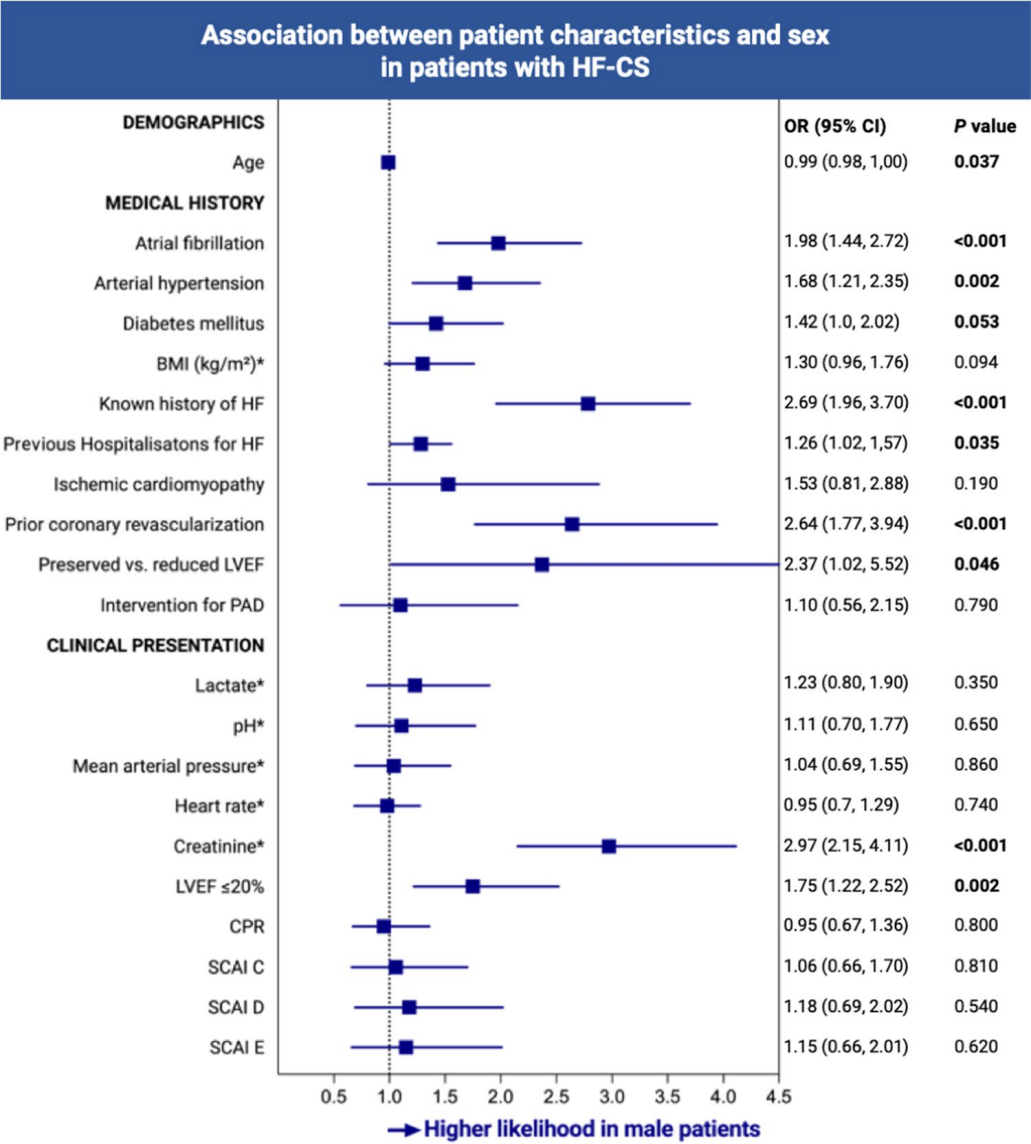
Association between patient characteristics and sex in patients with heart failure–related cardiogenic shock. To investigate differences in clinical characteristics during the index event between women and men with HF-CS, multivariable mixed effects logistic regression models were fitted, adjusted for age, SCAI class, lactate, pH, prior CPR, and mechanical ventilation. BMI, body mass index; CI, confidence interval; CPR, cardiopulmonary resuscitation; HF-CS, heart failure–related cardiogenic shock; LVEF, left ventricular ejection fraction; OR, odds ratio; PAD, peripheral artery disease; SCAI, Society for Cardiovascular Angiography & Interventions. An asterisk denotes dichotomized by median

At the index event, when comparing parameters consistent with CS severity, both groups exhibited similar values for blood pressure, heart rate, and lactate measurements. However, women were less likely to present with renal dysfunction (baseline creatinine dichotomized by median: men vs. women, OR 2.97, 95% CI 2.15–4.11, *p* < 0.001), and also less likely to present with a severely depressed LVEF (OR 1.75, 95% CI 1.22–2.52, *p* = 0.002). Detailed associations of sex with comorbidities, clinical presentation, and CS severity in patients with HF-CS are depicted in Fig. 1.

### Sex-related differences in treatments of HF-CS

**Fig. 2:**
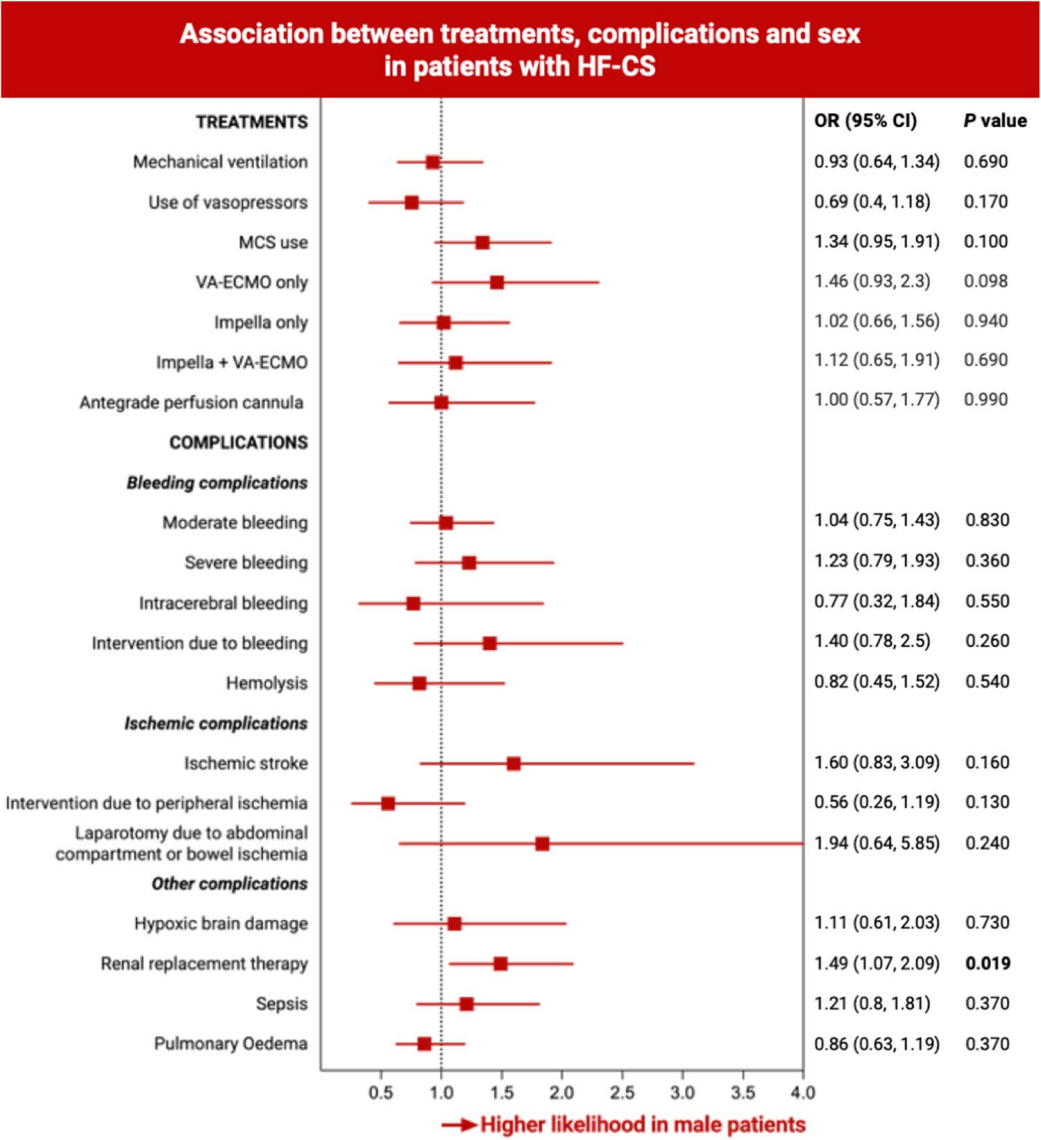
Association between treatments, complications, and sex in patients with heart failure–related cardiogenic shock. To investigate differences in treatment modalities and complications between women and men with HF-CS, multivariable mixed effects logistic regression models were fitted, adjusted for age, SCAI class, lactate, pH, prior CPR, and mechanical ventilation. CI, confidence interval; HF-CS, heart failure–related cardiogenic shock; MCS, mechanical circulatory support; OR, odds ratio; VA-ECMO, veno-arterial extracorporeal membrane oxygenation

Among patients with HF-CS, 893 (86.8%) received vasopressors, and 406 (39.4%) MCS. Crude MSC usage rates indicated a lower utilization of VA-ECMO in women as compared to men (12% vs. 18%, *p* = 0.031, Table 1). However, after adjusting for relevant confounders, no significant disparities were observed between the groups (mechanical ventilation, OR 0.93, 95% CI 0.64–1.34, *p* = 0.69; use of vasopressors, OR 0.69, 95% CI 0.40–1.18, *p* = 0.17; any MCS, OR 1.34, 95% CI 0.95–1.91, *p* = 0.097; Impella only, OR 1.02, 95% CI 0.66–1.56, *p* = 0.94; VA-ECMO only, OR 1.46, 95% CI 0.93–2.3, *p* = 0.098; Impella + VA-ECMO, OR 1.12, 95% CI 0.65–1.91, *p* = 0.69, Fig. 2). In this study cohort, none of the patients received treatment with an intra-aortic balloon pump.

### Sex-related differences in shock severity and end-organ failure in HF-CS

There was no significant association observed between sex and SCAI CS stages (women vs. men: OR 1.06, 95% CI 0.66–1.70, *p* = 0.81 for SCAI C; OR 1.18, 95% CI 0.69–2.02, *p* = 0.54 for SCAI D; OR 1.15, 95% CI 0.66–2.01, *p* = 0.62 for SCAI E, with SCAI B as reference), as depicted in Fig. 1.

**Fig. 3:**
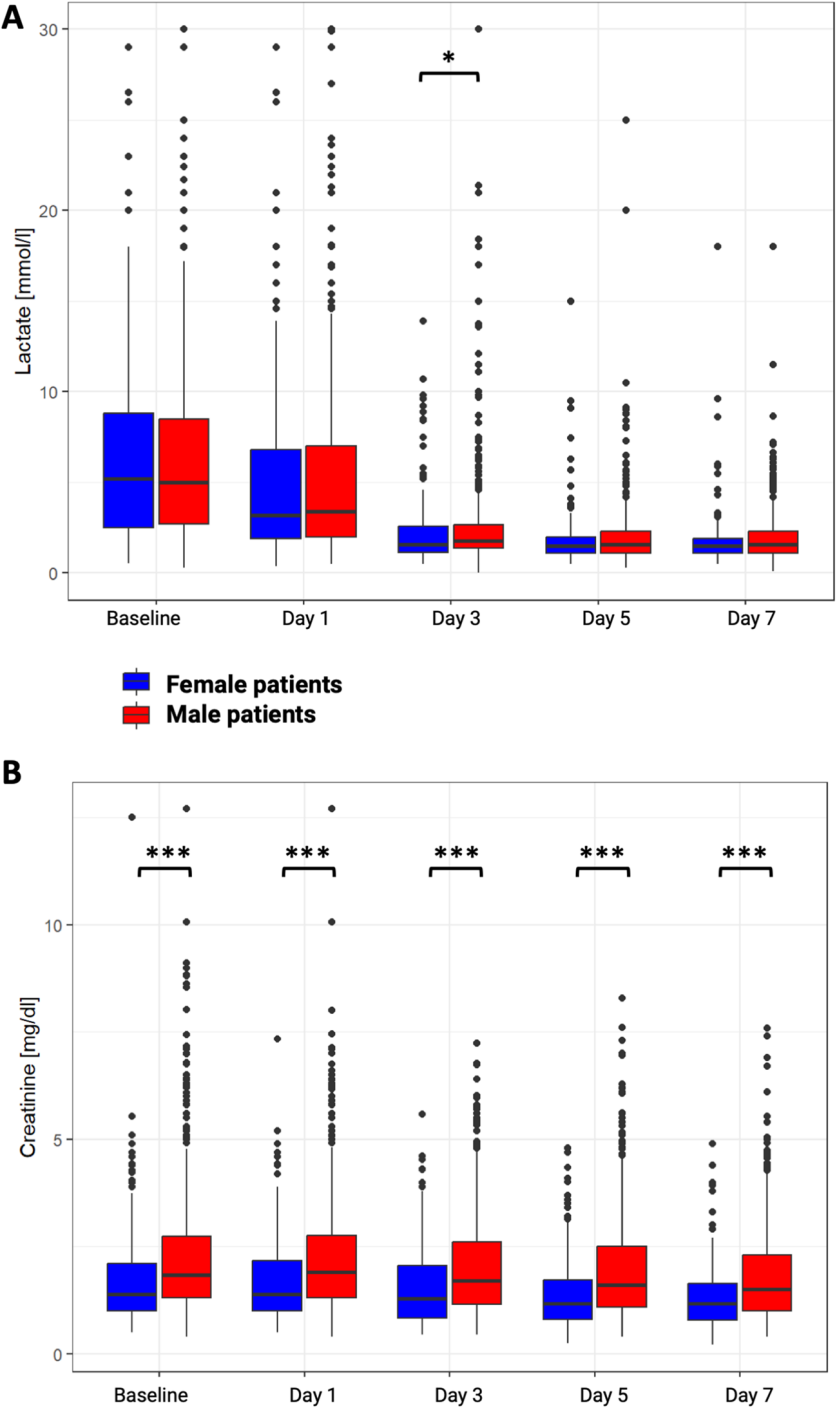
Lactate and creatinine trajectories from baseline to day 7 in women and men with heart failure–related cardiogenic shock. To evaluate shock dynamics following the index event, trajectories of lactate levels (**A**), as an indicator of shock severity, and creatinine levels (**B**), as a marker of end-organ damage, in female and male patients diagnosed with HF-CS over a period of 7 days. *p < 0.05; ***p < 0.001

Laboratory markers assessing hypoperfusion (lactate) and end-organ damage (creatinine), from baseline to day 7, were examined and stratified by sex (Fig. 3). In both groups, favorable lactate clearance was observed over the initial 7-day period. At the index event of HF-CS, a comparable high lactate level was observed between women and men (lactate women vs. men, 5.2 mmol/l vs. 5.0 mmol/l, *p* = 0.91). However, on day 3, a slightly faster lactate clearance was detected in women (lactate women vs. men, 1.6 mmol/l vs. 1.8 mmol/l, *p* = 0.017), with a persistent trend observed until day 7 (Fig. 3A). In terms of creatinine trajectories, a significantly lower concentration of creatinine was observed in women compared to men throughout the entire duration of 7 days (creatinine women vs. men: for baseline 1.4 vs. 1.8 mg/dl, *p* < 0.0001; for day 1 1.4.1 vs. 1.9 mg/dl, *p* < 0.0001; for day 3 1.3 vs. 1.7 mg/dl, *p* < 0.0001; for day 5 1.2 vs. 1.6 mg/dl, *p* < 0.0001; for day 7 1.2 vs. 1.5 mg/dl, *p* < 0.0001, Fig. 3B).

### Outcome and complications

**Fig. 4:**
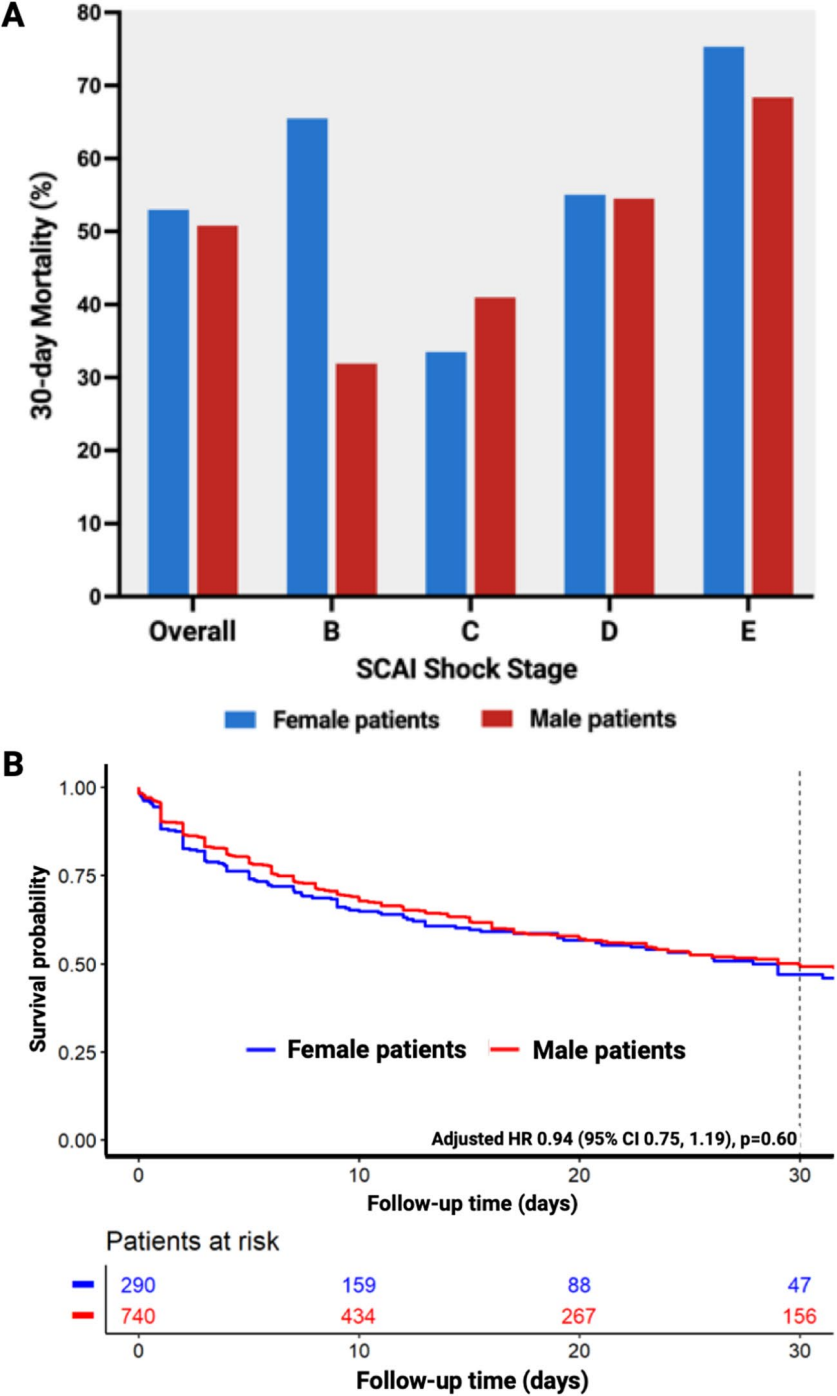
Mortality in patients with heart failure–related cardiogenic shock stratified by sex. **A** Mortality stratified by SCAI CS Staging in women vs. men with heart failure–related cardiogenic shock. **B** Kaplan–Meier curves comparing women vs. men with heart failure–related cardiogenic shock. CI, confidence interval; HR, hazard ratio; SCAI, Society for Cardiovascular Angiography & Interventions

Among patients with HF-CS in this registry, a total of 445 patients died within the 30-day follow-up period, resulting in a crude 30-day mortality rate of 51.4%. Women exhibited a 30-day mortality rate of 53.0%, whereas men had a nearly similar mortality rate of 50.8% (Fig. 4B). The 30-day mortality rates for women and men, stratified by SCAI CS staging at the index event, are illustrated in Fig. 4A. After adjustment for relevant confounders (age, SCAI class, lactate, pH, prior CPR, mechanical ventilation), women were associated with a similar probability of 30-day all-cause mortality compared to men, with a hazard ratio of 0.94 (95% CI 0.75–1.19, *p* = 0.60).

In women with HF-CS, renal replacement therapy was less frequently observed compared to men (23.8% vs. 34.4%, *p* = 0.001), even after adjustment for relevant confounders (HR 1.49, 95% CI 1.07–2.09, *p* = 0.019). The probability for other complications such as bleeding, ischemic complications, or sepsis was comparable between the two study groups, even after adjustment for relevant confounders (Table 2, Fig. 2).

**Table 2:** Complications stratified by sex

	Female patients (*N* = 290)	Male patients (*N* = 740)	*p* value
Bleeding complications			
Moderate bleeding	89 (30.9)	249 (33.8)	0.42
Severe bleeding	34 (11.8)	114 (15.4)	0.14
Intracerebral bleeding	9 (3.2)	19 (2.7)	0.67
Hemorrhagic stroke	1 (0.4)	6 (0.8)	0.68
Intervention due to bleeding	17 (5.9)	69 (9.3)	0.079
Hemolysis	18 (6.2)	47 (6.4)	1.00
Ischemic complications			
Ischemic stroke	15 (5.3)	54 (7.6)	0.22
Intervention due to access-site-related ischemia	13 (4.5)	23 (3.1)	0.35
Laparotomy due to abdominal compartment or bowel ischemia	4 (1.4)	20 (2.7)	0.26
Other complications			
Hypoxic brain damage	20 (7.1)	51 (7.2)	1.00
Renal replacement therapy	69 (23.8)	253 (34.3)	0.001
Sepsis	42 (14.5)	138 (18.7)	0.12

Continuous variables are shown as a median (25th, 75th percentile), binary variables as absolute and relative frequencies, the *p* value given is calculated for continuous variables by Mann–Whitney test or binary variables by Fisher’s exact test

## Discussion

In this large multicenter, international study of patients with HF-CS, compared to men, women tended to be older, exhibited fewer cardiovascular risk factors, and were more likely to present with de novo HF (e.g., lower prevalence of acute-on-chronic HF). Furthermore, women were less likely to present with a severely depressed LVEF or with renal dysfunction, resulting in a decreased requirement for dialysis. Nevertheless, use of treatments was comparable between women and men, and even after adjusting for relevant confounders, women and men faced similarly high mortality risk.

### Sex-related differences in clinical presentation in HF-CS

Recent research findings indicate that a substantial proportion of patients with CS may be attributed to HF, independent of AMI as the underlying etiology, with a short-term mortality of around 50% [4–8, 24–26]. The heterogeneity of the underlying pathology in HF-CS poses significant clinical challenges in terms of risk-stratifying patients and tailoring CS treatments [14, 15]. Among the various factors that could contribute to this heterogeneity, sex potentially may be a significant factor in patients with HF-CS and thus was further investigated in this study.

In this context, we observed that sex influenced demographics and clinical presentation in patients with HF-CS. Specifically, women tended to be older than men and had a lower prevalence of typical cardiovascular risk factors such as diabetes and hypertension, as well as cardiac comorbidities like atrial fibrillation and renal dysfunction. As known from studies in chronic heart failure, female patients were less frequently treated with guideline-directed medical therapy [27]. Furthermore, our results indicated that women with HF-CS were less likely to present with a severely depressed LVEF, were more likely to present with de novo as compared to acute-on-chronic HF-CS, and were less likely to have had prior hospitalizations due to HF-CS. Although direct comparisons are limited, these findings contrast prior observations in patients with AMI-CS. These suggested that women had a worse cardiovascular risk profile compared to men, but consistently demonstrated an association between female sex and advanced age in CS [17, 18, 28, 29].

Overall, the observation that women are more likely to present with a higher LVEF, without prevalent HF, and with fewer comorbidities suggests the presence of different disease mechanisms in women vs. men presenting with HF-CS. Consequently, this might then also be translated into different treatment algorithms for women vs. men, e.g., introducing sex-tailored treatment strategies to the field of CS.

### Sex-related differences in shock severity and end-organ failure in HF-CS

Evaluation of several parameters of CS severity, including SCAI CS risk class, indicated comparable clinical profiles between women and men presenting with HF-CS. However, previous research has highlighted the importance of short-term lactate kinetics as a prognostic indicator and a marker for end-organ failure in CS [30]. In our study, we observed a slightly faster clearance of serum lactate in women within the first few days after presentation, with a persistent trend over subsequent days. Additionally, we observed a significantly shorter duration of renal dysfunction in women compared to men over time, potentially indicating less subclinical end-organ damage. These findings suggest that women with HF-CS, although initially presenting with comparable CS severity, may have inherent physiological advantages that enable them to achieve quicker recovery from shock onset. This could be attributed to the higher rate of de novo HF-CS in women, e.g., lesser (sub-)clinical end-organ damage due to pre-existing HF, but also to their lower comorbidity burden [22]. Moreover, the role of systemic inflammation in patients with HF-CS, as well as their intersexual differences, is currently unclear and warrants further investigation [31]. Although further research is needed to elucidate the exact underlying mechanisms and to confirm these data, our observations might be used as a first step towards sex-tailored treatment strategies.

### Sex-related differences in treatments of HF-CS

Currently, there is limited evidence for the tailored use of inotropics, vasopressors, MCS, and cause-specific therapeutic interventions in the management of HF-CS. The use of catecholamines in the treatment of CS remains the subject of debate, although there is a growing consensus on their short-term administration to stabilize patients for further therapeutic strategies [32–34]. It is noteworthy that most randomized controlled trials excluded patients with HF-CS (without AMI-CS), leading to a dearth of evidence-based therapeutic approaches [35–37]. The potential role of MCS devices to stabilize hemodynamic aberrations and bridge to native heart recovery is a promising option, supported by findings of a prior propensity-matched analysis from our registry [21]. However, the presence of complications with MCS remains a noteworthy concern [4, 21, 38–42].

In this study, we observed that vasopressors were administered to over 86% of the patients with HF-CS. Interestingly, despite limited evidence on the use of MCS in HF-CS, 39% of the patients in our cohort required MCS during their hospitalization. Importantly, there was no association between sex and the use of treatment modalities such as vasopressors, mechanical ventilation, and MCS. While we observed that women received slightly more ECMO therapy compared to men in this study, the utilization of MCS, including various MCS devices, was similar after adjusting for relevant confounders in women and men with HF-CS. These results contrast with previous studies in AMI-CS, where women were less likely to undergo MCS therapy [16, 18, 43, 44].

As indicated above, differences in clinical presentation in women vs. men (e.g., fewer comorbidities, lower rates of preexisting HF, and better LVEF) indicate sex-specific peculiarities in the pathomechanisms of CS. Most importantly, the higher LVEF observed in women suggests that they might respond differently to therapies targeting ventricular function. MCS, which specifically addresses this issue by providing cardiac output support until native heart recovery or durable replacement therapy, may be less effective in this subgroup, where depressed LVEF might not be the main problem. This suggests a more restrictive use of MCS in women with HF-CS, especially as the risk of MCS-related complications is similar, if not higher, in women vs. men. In addition, there are difficulties with regard to MCS access due to smaller vessels in women. Access size improvements are urgently needed to bridge the sex gap and allow women equal opportunities to benefit from these technologies while minimizing vascular complications. Ultimately, the decision to initiate MCS should be based on a comprehensive assessment of each patient’s clinical condition and hemodynamic profile, but most likely should also include the patients sex and the expected response to MCS use [45, 46].

### Association between sex and 30-day all-cause mortality

In this study, despite women presenting with fewer cardiovascular risk factors, lesser comorbidities, a lower prevalence of pre-existing HF, better renal function, and higher LVEF as compared to men, 30-day all-cause mortality rate was comparable in women vs. men. Previous studies have indicated higher mortality rates among women compared to men in the context of CS and have often been attributed to factors such as older age, a greater burden of comorbidities, and a lower likelihood of early revascularization and MCS use. However, in this study, although they were more likely to be older, women tended to have less cardiovascular risk factors and comorbidities, and use of treatments was comparable in women vs. men, which might explain the lack of a sex-specific mortality risk. Also, previous studies on CS were mainly conducted in patients with AMI as the underlying etiology, and differences in pathomechanisms between AMI and HF as the cause of CS might contribute to explain the differences in mortality risks. Ultimately, based on the prior observation of differences in clinical presentation between women and men with HF-CS, it is tempting to speculate if the “true” mortality risk of women presenting with HF-CS might be even lower than in men if sex-specific treatment strategies had been used. Severe vascular complications, with subsequent interventions due to access-site-related ischemia or bleeding, may occur more frequently in women due to smaller vessel size. Access options for modern MCS devices, especially the still large ECMO cannulas, should be adapted and urgently improved to address anatomical differences in women, aiming to further enhance the risk–benefit ratio in women with HF-CS.

### Limitations

The data used in this study were non-randomized, preventing us from establishing causal relationships between risk predictors and outcomes. Additionally, the assessment of patient characteristics may have been influenced by subjective judgments, particularly in challenging clinical environments such as intensive care units, emergency departments, or catheterization laboratories. Furthermore, this cohort did not collect invasive hemodynamic parameters, which could have provided additional insights and verification of sex-related differences. Although data were gathered from different hospitals in various countries, it is important to note that these hospitals were large tertiary care centers with significant experience in managing CS and utilizing MCS. This may have resulted in a higher use of MCS and a higher prevalence of severe CS in the cohort. It should be acknowledged that the use of MCS is a selective process, often favoring patients with higher physiological reserve. As a result, the generalizability of these findings may be limited, and there is a pressing need for randomized controlled trials specifically focusing on patients with HF-CS.

Another crucial aspect to consider is the potential for selective “healthiness” among women within the context of this study. It is conceivable that women with comparable comorbidities and preexisting HF might not be identified as HF-CS patients or included in the study’s registry. This potential for selection bias may contribute to the observed disparities in clinical characteristics and outcomes between sex.

### Clinical implications

These findings suggest that healthcare providers should be aware of potential differences in clinical presentation and comorbidities between women and men with HF-CS. The higher LVEF, lower rates of preexisting heart failure, fewer comorbidities, and lower incidence of renal failure as well as faster lactate clearance observed in women with HF-CS indicate that they may follow a distinct trajectory and may benefit from tailored management strategies to optimize outcomes. One potential approach could be to adopt a more restrictive utilization of MCS in women. Given their higher inherent potential for stabilization, a less liberal MCS strategy may be considered.

Importantly, despite these disparities, both female and male patients with HF-CS face similar high mortality risks, emphasizing the need for adequate interventions to improve outcomes for all patients with this critically ill population. Further research is warranted to better understand the underlying mechanisms contributing to develop targeted interventions and sex-tailored treatment strategies that address the unique needs of female and male patients with HF-CS.

## Conclusion

In this large, international study of patients with HF-CS, compared to men, women tended to be older, but had fewer cardiovascular risk factors and were less likely to present with prevalent HF, a severely depressed LVEF, or with renal dysfunction. Also, lactate clearance was faster and end-organ damage lower in women vs. men.

Nevertheless, even with comparable use of treatments in women vs. men and after adjusting for relevant confounders, mortality risk was comparable.

Further research is necessary to evaluate if sex-tailored treatment, e.g., accounting for the differences in clinical presentation, might improve outcomes in HF-CS.

## Data Availability

The study findings are based on primary data, which can be obtained from the corresponding author upon a reasonable request.

## Abbreviations

AMI: Acute myocardial infarction
CPR: Cardiopulmonary resuscitation
CS: Cardiogenic shock
HF: Heart failure
HF-CS: Heart failure–related cardiogenic shock
LVEF: Left ventricular ejection fraction
MCS: Mechanical circulatory support
SCAI: Society for Cardiovascular Angiography & Interventions
VA-ECMO: Veno-arterial extracorporeal membrane oxygenation
